# Supplementary material for: Exploring the Sources of Stress and Coping Strategies of Turkish Preschool Teachers
Source: Behav Sci (Basel). 2024 Jan 16;14(1):59. doi: 10.3390/bs14010059 (PMC10813605; doi:10.3390/bs14010059)
Supplement: Supplementary file 1 [file behavsci-14-00059-s001.zip › behavsci-2782043-supplementary.pdf]

SUPPLEMENTARY FILES

Table S1. Information about the participants

Teachers	The Institution	Age group receiving Education	Institution	Gender	Professional Seniority (Years)	Working Time	Availability of a Helper in the Class	Inclusion Student	Branch of the School Principal
T1	Private	0–3 years	Preschool	Female	4–7 years	Full day	Yes	None	Other
T2	Private	0–3 years	Preschool	Female	4–7 years	Full day	Yes	None	Other
T3	Private	0–3 years	Preschool	Female	1–3 years	Full day	Yes	None	Preschool
T4	Private	0–3 years	Preschool	Female	4–7 years	Full day	Yes	None	Preschool
T5	Private	0–3 years	Preschool	Female	1–3 years	Full day	Yes	Yes	Preschool
T6	Private	0–3 years	Preschool	Female	4–7 years	Full day	Yes	None	Preschool
T7	Private	3–4 years	Preschool	Female	4–7 years	Full day	Yes	None	Other
T8	Private	3–4 years	Preschool	Female	1–3 years	Full day	Yes	None	Other
T9	Private	3–4 years	Preschool	Female	12–15 years	Full day	Yes	Yes	Preschool
T10	Private	3–4 years	Preschool	Female	1–3 years	Full day	Yes	Yes	Preschool
T11	Private	3–4 years	Preschool	Female	1–3 years	Full day	Yes	Yes	Preschool
T12	Private	3–4 years	Preschool	Female	4–7 years	Full day	Yes	None	Other
T13	Private	4–6 years	Preschool	Female	12–15 years	Full day	Yes	None	Preschool
T14	Private	4–6 years	Preschool	Female	1–3 years	Full day	Yes	None	Preschool
T15	Private	4–6 years	Preschool	Female	1–3 years	Full day	Yes	None	Preschool
T16	Private	4–6 years	Preschool	Female	1–3 years	Full day	Yes	None	Preschool
T17	Private	4–6 years	Preschool	Female	4–7 years	Full day	Yes	None	Other
T18	Private	4–6 years	Preschool	Female	1–3 years	Full day	Yes	None	Preschool
T19	State	4–6 years	Preschool	Female	4–7 years	Half day	None	None	Preschool
T20	State	3–4 years	Preschool	Female	4–7 years	Half day	None	None	Preschool
T21	State	3–4 years	Preschool	Female	12–15 years	Half day	None	None	Other
T22	State	3–4 years	Preschool	Female	4–7 years	Half day	None	None	Preschool
T23	State	3–4 years	Preschool	Female	4–7 years	Half day	None	None	Preschool
T24	State	3–4 years	Preschool	Female	4–7 years	Half day	Yes	None	Preschool
T25	State	3–4 years	Preschool	Female	8–11 years	Half day	Yes	None	Preschool
T26	State	4–6 years	Preschool	Female	8–11 years	Half day	Yes	None	Preschool
T27	State	4–6 years	Preschool	Female	8–11 years	Half day	Yes	Yes	Preschool
T28	State	4–6 years	Preschool	Female	8–11 years	Half day	Yes	None	Preschool
T29	State	4–6 years	Preschool	Female	8–11 years	Half day	Yes	None	Preschool
T30	State	4–6 years	Preschool	Female	8–11 years	Half day	Yes	Yes	Preschool
T31	State	4–6 years	Kindergarten	Female	8–11 years	Half day	Yes	Yes	Other
T32	State	4–6 years	Kindergarten	Female	8–11 years	Half day	None	None	Other
T33	State	4–6 years	Kindergarten	Female	16–19 years	Half day	None	None	Other
T34	State	4–6 years	Kindergarten	Female	1–3 years	Half day	None	None	Preschool
T35	State	4–6 years	Kindergarten	Female	12–15 years	Half day	None	None	Other
T36	State	4–6 years	Kindergarten	Female	4–7 years	Half day	None	None	Other

Note: Table 1 contains demographic information of the participants.

Table S2. COREQ results

Topic	Item No.	Guide Questions/Description	Reported on Page No.
**Domain 1: Research team and reflexivity**			
Personal characteristics			
Interviewer/facilitator	1	Which author/s conducted the interview or focus group?	Page No. 6
Credentials	2	What were the researcher's credentials? E.g. PhD, MD	PhD, Page No. 1
Occupation	3	What was their occupation at the time of the study?	Page No. 1
Gender	4	Was the researcher male or female?	Male, Page No. 1
Experience and training	5	What experience or training did the researcher have?	Page No. 6
Relationship with participants			
Relationship established	6	Was a relationship established prior to study commencement?	Page No. 6
Participant knowledge of the interviewer	7	What did the participants know about the researcher? e.g. personal goals, reasons for doing the research	Page No. 6
Interviewer characteristics	8	What characteristics were reported about the interviewer/facilitator? e.g. Bias, assumptions, reasons, and interests in the research topic	None
**Domain 2: Study design**			
Theoretical framework			
Methodological orientation and Theory	9	What methodological orientation was stated to underpin the study? e.g. grounded theory, discourse analysis, ethnography, phenomenology, content analysis	Page No. 4
Participant selection			
Sampling	10	How were participants selected? e.g. purposive, convenience, consecutive, snowball	Page No. 4-5
Method of approach	11	How were participants approached? e.g. face-to-face, telephone, mail, email	Page No. 6
Sample size	12	How many participants were in the study?	Page No. 5
Non-participation	13	How many people refused to participate or dropped out? Reasons?	Page No. 5
Setting			
Setting of data collection	14	Where was the data collected? e.g. home, clinic, workplace	Page No. 6
Presence of non-participants	15	Was anyone else present besides the participants and researchers?	None
Description of sample	16	What are the important characteristics of the sample? e.g. demographic data, date	Page No. 5
Data collection			
Interview guide	17	Were questions, prompts, guides provided by the authors? Was it pilot tested?	Page No. 6
Repeat interviews	18	Were repeat inter views carried out? If yes, how many?	None, Page No. 6
Audio/visual recording	19	Did the research use audio or visual recording to collect the data?	Page No. 6
Field notes	20	Were field notes made during and/or after the interview or focus group?	None, Page No. 6
Duration	21	What was the duration of the inter views or focus group?	Page No. 6
Data saturation	22	Was data saturation discussed?	Page No. 6
Transcripts returned	23	Were transcripts returned to participants for comment and/or correction	Page No. 6
**Domain 3: Analysis and findings**			
Data analysis			

Number of data coders	24	How many data coders coded the data?	Page No. 6
Description of the coding tree	25	Did authors provide a description of the coding tree?	Page No. 6
Derivation of themes	26	Were themes identified in advance or derived from the data?	Page No. 6
Software	27	What software, if applicable, was used to manage the data?	None, Page No. 6
Participant checking	28	Did participants provide feedback on the findings?	None, Page No. 6
Reporting			
Quotations presented	29	Were participant quotations presented to illustrate the themes/findings? Was each quotation identified? e.g. participant number	Page No. 7-11
Data and findings consistent	30	Was there consistency between the data presented and the findings?	Page No. 7-11
Clarity of major themes	31	Were major themes clearly presented in the findings?	Page No. 7
Clarity of minor themes	32	Is there a description of diverse cases or discussion of minor themes?	Page No. 11-14

Form S1.

INTERVIEW FORM/POESEDURE

Dear preschool teacher,
This interview is scheduled as part of a research endeavor entitled "An Investigation into Stress Sources and Coping Mechanisms among Preschool Teachers." Your perspectives will be treated with utmost confidentiality, solely employed for research purposes, and subjected to anonymization. Participation is voluntary, and you retain the right to withdraw from the interview at any stage of the data collection process. Our conversation will be documented via a voice recorder; Your confirmation of this recording process is imperative. Alternatively, you should prefer not to have your voice recorded, I will resort to noting our discussion. Your commitment to this process is greatly appreciated.

Doç. Dr. Sinan KOÇYİĞİT
Doç. Dr. Türker SEZER

Meeting date:
Meeting time:
Introduction:
The researcher introduces himself. Then asks questions to the participant.
Let us commence by inviting you to provide an introduction about yourself.
Subsequently, I will be posing inquiries designed to elicit descriptive insights about your current professional background.
What is your professional seniority?
What are the characteristics of the institution you work for? (public/private)
Can you tell us about your working hours? (full day/half day)
What is the number and age group of children in your class?
Do you have a helper in the classroom?
Do you have an inclusion student in your class?
What is your school principal's area of expertise?
Thank you for the information you provided. Now, I move on to asking you the questions that form the framework of our research. Please do not rush when answering the questions. Try to give examples from your experiences.

MAIN INTERVIEW QUESTIONS

1. What/what situations do you see as sources of stress while teaching? From where? Please explain.
2. In what situations do you experience stress as an educator?
a) Think about times of greatest stress. Can you describe these times?
b) Why do you think these times create stress?
3. How do you deal with stressors? What methods do you use? Please explain.

Is there anything you want to add?
Our meeting is over. Could you please listen to the conversation I recorded?
Do you approve of the recording and what you said/statements?
sincerely thank you for your participation. You can contact me when you want information about the results of the research.
Interview ending time:

Form S2.

INTERVIEW SCHEDULE

	Private school, teachers of 0-3-year-old children (nuresry)	Private School, teachers of 3-4-year-old children (preschool)	Private School, teachers of 4-6 years old children (preschool)	Public School, teachers of 3-4-year-old children (preschool)	Public School, teachers of 4-6-year-old children (preschool)	Public School, teachers of 4-6-year-old children (kindergarten)
Date	20-22 May	27-29 May	03-05 June	10-12 June	17-19 June	24-26 June
meeting place	Teachers' room	Teachers' room	Teachers' room	Teachers' room	Teachers' room	Teachers' room
